# Dynamics and Structure in Cell Signaling Networks: Off-State Stability and Dynamically Positive Cycles

**DOI:** 10.1371/journal.pone.0057653

**Published:** 2013-03-08

**Authors:** Dániel Kondor, Gábor Vattay

**Affiliations:** Department of Physics of Complex Systems, Eötvös Loránd University, Budapest, Hungary; University of Ulm, Germany

## Abstract

The signaling system is a fundamental part of the cell, as it regulates essential functions including growth, differentiation, protein synthesis, and apoptosis. A malfunction in this subsystem can disrupt the cell significantly, and is believed to be involved in certain diseases, with cancer being a very important example. While the information available about intracellular signaling networks is constantly growing, and the network topology is actively being analyzed, the modeling of the dynamics of such a system faces difficulties due to the vast number of parameters, which can prove hard to estimate correctly. As the functioning of the signaling system depends on the parameters in a complex way, being able to make general statements based solely on the network topology could be especially appealing. We study a general kinetic model of the signaling system, giving results for the asymptotic behavior of the system in the case of a network with only activatory interactions. We also investigate the possible generalization of our results for the case of a more general model including inhibitory interactions too. We find that feedback cycles made up entirely of activatory interactions (which we call *dynamically positive*) are especially important, as their properties determine whether the system has a stable signal-off state, which is desirable in many situations to avoid autoactivation due to a noisy environment. To test our results, we investigate the network topology in the Signalink database, and find that the human signaling network indeed has only significantly few dynamically positive cycles, which agrees well with our theoretical arguments.

## Introduction

Biochemical interactions in a cell form complex networks with its elements carrying out many different tasks [1]. An important part is the signal transduction system which allows the cell to adapt to the environment. The working of this signaling system has been actively studied, both theoretically and experimentally, with databases now being available with growing information about the specific proteins making up the signal transduction network and their interactions [2]–[6]. As the signaling system is an essential part of the cell, a disruption in its functioning is believed to be a significant factor in many diseases, especially in cancer [7]–[9]. Thus, a better understanding of the signaling system will possibly allow more effective treatments. Also, from a theoretical point of view, the study of cell signaling can reveal how biochemical interactions are able to form a complex self-organizing system, capable of processing information in a noisy environment.

The signaling system is naturally modeled as a directed graph: the nodes are the protein species possibly present in a cell, and an edge points from node 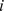 to node 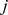 if protein species 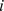 affects protein species 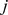. A kinetic model is usually a set of ordinary differential equations where the independent variables represent the concentration of the possible states of the proteins. An edge in the graph implies that the some of the variables associated with protein 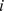 is present on the right-hand-side of the equations concerning some of the variables associated with protein 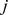.

In this paper we investigate the general mathematical model introduced by Heinrich et al. [10], [11]. We follow the simple hypothesis that activation in the signaling system should only occur as a result of an external or internal signal (e.g. a receptor is activated) [11], [12]. While in some cases this is not true (a signal only tunes the output [13]), this might prove as a correct basic principle for the part of the system which has to respond to the stimuli. Mathematically, this means that the *signal-off* state of the dynamical system describing the signaling network has to be stable and also attractive in the absence of stimulation so that the system will relax to it, and will not be spontaneously activated by any noise present [12].

The original model of Heinrich et al. includes only positive interactions: a protein species activating other proteins. In this case, we identify the global attractor of the system, which is reached in the presence of any constant external inputs. The addition of inhibitory interactions gives rise to more complex behavior generally, but the property of whether the network has a stable *signal-off* state is preserved. To assess our theoretical arguments, we investigate the human signaling network found in the Signalink database [4], and find that having a stable *signal-off* state indeed seems to be an important factor shaping the network topology.

## Results

### A Network Model with Positive Links

A possible model of the signaling network is that of Heinrich et al. [10], [11]. In this model, each protein has two possible states (active and inactive, activation is achieved via phosphorylation), with only proteins in their active state interacting with others. A protein can become activated by another protein or by a stimulated receptor which is considered to be the external input driving the system. Deactivation occurs via phosphatases, which are considered to work independently of the rest of the system. This model is general in the sense that multiple interconnected signaling pathways can be included in one network, while it contains only simple activating interactions. This means that this model is targeted at the propagation of some signal (primarily considering, but not restricted to an external stimulus getting into the cell’s nucleus) not including some advanced features like adaptation which require a more specialized treatment [6], [13].

### The Equations Considered

Using mass action kinetics, the system can be described by the following ordinary differential equations:
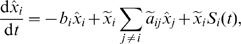
(1)where 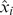 and 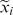 are the concentrations of the 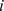-th protein in the active and inactive state respectively. The 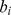 constants describe the deactivation of the 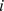-th protein with its rate being independent of the other protein concentrations. The elements of the matrix 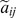 describe the activation of protein 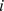 by protein 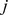. We assume that all 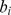 are positive, all 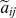 are nonnegative and the diagonal elements are all zero: 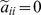. The term 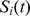 is an external signal affecting the 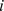-th protein, with 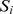 being positive for proteins receiving input, and zero for all others. We will assume that the external signal is constant or changes on a time scale significantly larger than the rest of the system, and write 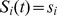. We also make the assumption that the total concentration of each protein species is constant: 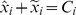, which allows us to write Eq. (1) in a dimensionless form:
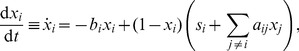
(2)where we defined 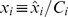 and 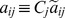. Note that now all 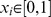. Also, the new matrix elements 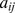 are scaled by the total protein concentration 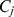, meaning that the change of total concentrations (e.g. by the synthesis of proteins) can be represented in this model by varying the elements of the matrix 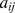. We chose not to include the effects of concentration changes in the model itself, but this allows the comparison of two systems where the 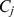 concentrations differ.

In the following, we will denote the vector with components 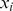 by 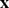. Throughout this paper we interpret relations between vectors component-wise, e.g. 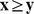 (or 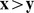) means that 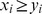 (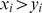 respectively) for all 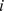.

If we have no external signal (all 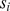 are zero), the *off-state* (when 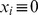 for all 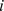) is always a stationary solution. In the special case, when the network contains no cycles (i.e. a cascade), the off-state is always stable and globally attractive; a finite-time signal has a response which relaxes to the off-state exponentially [10]. If the network contains cycles, the off-state can be unstable and a nonzero solution of Eq. (2) can persist even in the absence of an external signal. Linear stability of the off-state with respect to the network topology was investigated by Kartal and Ebenhöh [12]. We now give the general attractor of this system in the presence of arbitrary constant inputs.

The relation of Eq. (2) with the interaction graph of the protein network is quite natural. An edge 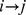 implies that 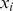 is present in the equation concerning 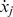. The 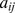 matrix in Eq. (2) can be considered as the appropriately weighted adjacency matrix of the interaction graph.

### The General Attractor

We can make a general statement about the solutions of Eq. (2) which hold for any possible combination of coefficients 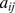 and 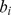 with any combination of stationary inputs 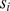. In this section we summarize our results. Proof of our statements is presented in the Methods section.

Eq. (2) always has exactly one stable stationary solution which is globally attractive with respect to the valid range of initial conditions 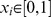, and may have other stationary solutions which are unstable. Despite that Eq. (2) describes a nonlinear system, no more complex behavior is possible; the system started from any initial condition which is not itself an unstable stationary solution relaxes to the unique stable stationary solution.

The main idea is that if there is a linearly stable stationary solution of Eq. (2), then a Lyapunov function can be used to show that it is attractive for any initial condition from the 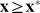 region. This implies that there can be no other stable stationary solutions as their basins of attraction would overlap. Considering the region where for at least one coordinate 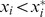, we use the properties of the Jacobian matrix 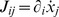 of the system to prove that regardless of the initial condition (except for a possible unstable stationary solution) the system will reach the 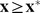 region where the Lyapunov function is applicable. Also, using the properties of the Jacobian matrix, we prove the existence of at least one linearly stable stationary solution.

Our results extend the those of Kartal and Ebenhöh [12]; considering the system described by Eq. (2), they examined linear stability and concluded that a linearly stable off-state is a significant property of real-world signaling networks. Using our results, it follows that a stable stationary solution is globally attractive; if the off state is linearly stable, the system will eventually return to it after any external signal has been shut off.

### Networks with Inhibitory Interactions

We examined the generalization of Eq. (2) to include inhibitory interactions. We considered the case when a protein in its activated state can deactivate other proteins, which can be described by the following equations:
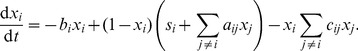
(3)


Here the elements of the 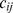 matrix describe the deactivation of protein 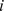 by protein 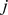. We assume that all 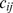 are nonnegative with the diagonal elements being zero. If we set all 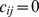, we get back the system (2). Computing the Jacobi matrix for the off-state (considering the case when the external signal 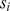 is zero), we get that it does not include the 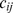 matrix. This means that the linear stability of the off-state depends only on the elements of the matrix 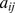 and the deactivation rate constants 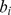. If the off-state for some particular system with only positive interactions is stable, then it remains stable if we add inhibitory interactions.

This is also true for the global attractive properties of the off-state; stability of the off-state implies that it is globally attractive even in the presence inhibitory interactions. The Lyapunov-function defined for the system with only positive interactions can be applied in this case too. Computing the derivative of this Lyapunov-function, we get that the terms containing the inhibitory interactions are nonpositive for all possible 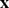, meaning that if a Lyapunov-function was valid in a network with positive interactions, then it remains valid after the addition of negative interactions (see the Methods section, especially Eqs. (8) and (15)).

A consequence of this property is that if we are interested in the stability of the off-state of a particular network, then we have to consider only the positive interactions between its nodes; we can restrict ourselves to the analysis of the subgraph which includes the positive links only. Behavior of the system in the absence of an external signal depends on the properties of the strongly connected components of that subgraph.

In the case when we have an external signal or the off-state is unstable, we cannot make a general statement about the solutions of Eq. (3). Numerical simulations show cases of monostability, multistability and periodic solutions, suggesting that the inclusion of inhibitory interactions might be necessary to account for a more complex behavior than which can result from Eq. (2), including a wide range of possible input–output patterns [14].

### General Two-state Networks

A more general model of the signaling network would have the following form:
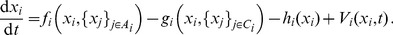
(4)


Eq. (4) is a possible generalization of Eq. (3). In this case too, every protein has two states, and only proteins in the active state can activate or deactivate other proteins. 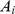 denotes the set of nodes which can activate node 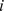 and 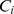 is the set of nodes which can deactivate node 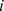 (i.e. there is a positive or negative edge between nodes 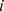 and 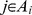 or 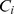). We assume, that for each 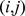 pair, node 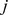 belongs to at most one of the sets 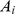 and 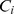. We also restrict the activation term 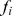 to be nonnegative and a monotonically decreasing function of 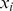, and monotonically increasing function of all 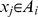. The deactivations term 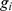 is also nonnegative, and a monotonically increasing function of 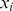 and all 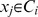. We also require the 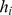 term describing the spontaneous deactivation of protein 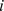 to be positive for all 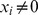, thus the concentration of the proteins relaxes to 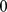 if there are no activating interactions. The term 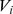 represents the external input on protein 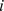. Of course, to ensure that the solutions stay in the 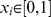 interval, we need to require that 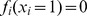, 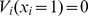, 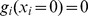 and 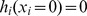 (these conditions will arise naturally if we derive Eq. (4) from some kinetic model where the total protein concentrations are preserved). Generally, we do not need the 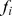, 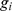, 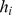, 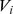 functions to be continuous or differentiable; if however, we want to carry out the linear stability analysis of a fixed point, then differentiability is required at least in some neighborhood of the fixed point. The most important consequence of the conditions prescribed on Eq. (4) is that the concentration of each protein relaxes to 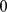 if it receives no activatory interactions or external input.

Giving the general solutions of a model like Eq. (4) can prove very difficult, with chaotic solutions possible in many cases [15]. This is why methods where a statement about the solutions of the model can be made based only on the network topology can prove very useful.

### Consequences of the Network Topology

The structure of Eq. (4) gives a very simple requirement for the system to have a nonzero solution in the absence of an external signal. We can notice that the only positive term on the right-hand-side of our equations is that describing activation, i.e. the term corresponding to positive links between proteins. If there are no cycles made up entirely of positive links (in the following sections we shall refer to these as *dynamically positive* cycles), then the system will relax to its off-state if there is no external signal. This is the generalization of the results we got for the specific model described by Eq. (3). The main assumption for this to be true is that in the absence of activatory interactions, each protein relaxes to its inactive state (e.g. via dephosphorylation by phosphatases). The requirement that a nonzero solution in the absence of an external signal can only persist if there are dynamically positive cycles holds true even if we include more complex processes, e.g. multiple phosphorylation.

On the other hand, a general network will possibly contain dynamically positive cycles. In this case, the stability of the off-state will depend on the nature of the functions involved in Eq. (4). Still, using the property that in the absence of activatory interactions, each protein relaxes to its inactive state, we can conclude that only the strongly connected components (SCCs) formed entirely by positive links (i.e. *dynamically positive SCC*s) are relevant. This means that instead of examining the properties of the whole network, we only have to evaluate the subnetworks which contain the points in each dynamically positive SCC. For the whole system to have a stable off-state, each of these subnetworks is required to have a stable off-state. This is the extension of the ideas in [12] to networks which include both activatory and inhibitory interactions. As every dynamically positive cycle can possibly give rise to an unstable off-state, having as few such cycles as possible (while retaining the biological functionality of the network) might be desirable to avoid an unwanted activation due to variations in parameter values (e.g. the total concentration of the signaling proteins depends on the expression level of the corresponding genes). In the following section, we test this hypothesis on the human signaling network in the Signalink database.

### Analysis of the Signalink Network

The Signalink database contains information about proteins and interactions present in three species: *Caenorhabditis elegans*, *Drosophila melanogaster* and *Homo sapiens*, which were manually collected from the literature [4], [16]. The interactions are labeled as either activation or inhibition. The networks of both *C. elegans* and *D. melanogaster* show a very simple topology with only a few cycles in them, while the human network has a largest SCC of 81 proteins and a total of 777041 cycles (see table 1). We carried out further analysis on the largest SCC of the human network, measuring the number of dynamically positive cycles. We compared the results with the same computed for random networks generated from the original database. We used three methods for network generation. In method (*a*) we exchanged the sign among randomly chosen pairs of edges, keeping the start and end nodes on both edges. Note that this method preserves the total number of positive or negative edges (if both edges had the same sign, no alteration was made). In method (*b*) we exchanged the endpoints of randomly chosen pairs of edges, thus also affecting the network topology (but in this case keeping the sign of the edges). Method (*c*) is an extension of method (*b*), where we also exchanged the sign of the chosen pair of edges with probability 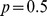.

**Table 1:** Signalink networks.

species	# of SCCs	# of proteins in thelargest SCC	# of cycles
H. sapiens	5	81	777041
D. melanogaster	2	5	7
C. elegans	3	4	4

Comparision of the signaling networks of the three species present in the Signalink database.

**Figure 1:**
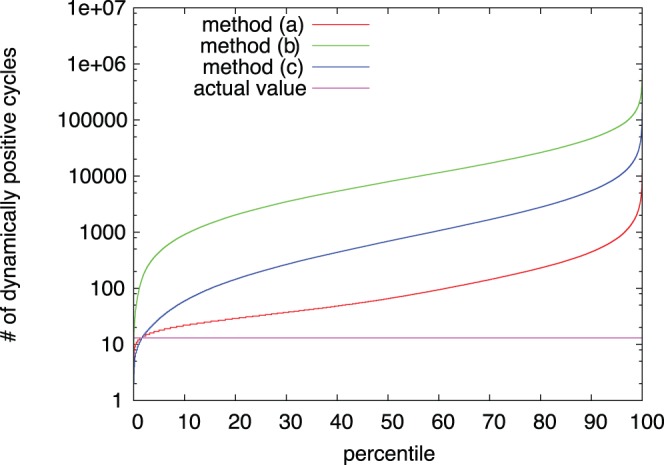
Percentile plot of dynamically positive cycles in random networks. The lines intersect with the actual value at 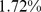 for method (*a*) (i.e. 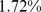 of all random networks generated had 13 or fewer dynamically positive cycles), 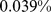 for method (*b*), and 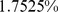 for method (*c*).

Based on our theoretical arguments, we expected the real network to have as few dynamically positive cycles as possible. With respect to the stability of the off-state, having no such cycles would be ideal, while this might not be acceptable as dynamically positive cycles could serve a specific purpose. The network in the Signalink database had 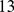 dynamically positive cycles. We compared this value with the number of such cycles in 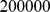 random graphs for each method, where each random graph was generated by making 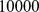 exchanges in the original network. For all three methods, the distribution of the number of dynamically positive cycles is fat-tailed; number of such cycles spans more than five orders of magnitude. The percentile plot of the measured distribution shows that the majority of the random graphs generated has more dynamically positive cycles than the actual value (see Fig. 1, pay attention to the logarithmic scaling of the 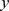-axis). The ratio of graphs having 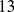 or less dynamically positive cycles is 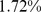 for method (*a*), 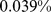 for method (*b*) and 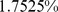 for method (*c*). This suggests that the number of dynamically positive cycles in the real network is significantly low. This implies, that the number of such cycles is indeed an important property of the signaling network, and while having some such cycles might not be avoidable, a small number is desirable.

From a theoretical view, the importance of dynamically positive cycles arises from two preliminary hypotheses; the assumption that each protein in the network relaxes to its inactive state if it does not receive activatory interactions; and the requirement that the signaling system has a stable off-state. Finding only a significantly small number of dynamically positive cycles in the human signaling network suggests that these hypotheses are valid for a large part of the system.

### Dynamically Positive Cycles in the Human Network

**Figure 2:**
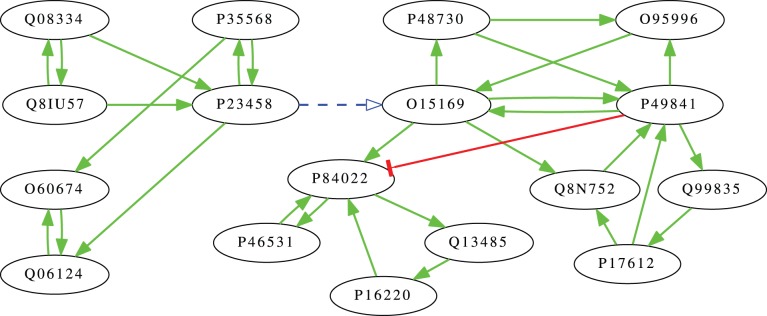
Dynamically positive cycles in the human network. The node labels are UniProt IDs, green arrows represent activatory interactions, while the red arrow is an inhibitory link. The dashed blue arrow between nodes P23458 and O15169 represents a path made entirely of activatory links.

In the case of the human signaling network in the Signalink database, we identified 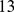 dynamically positive cycles, which form 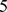 SCCs (three of which consist of only two nodes each). These 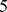 SCCs are all connected in one way with path made up of positive interactions. Table 2 lists the Uniprot IDs [17] of the proteins involved in them, while Fig. 2 shows the interactions between them.

**Table 2:** Dynamicaly positive SCCs of the human network.

#	proteins in SCC		# of proteins reachable through positive edges
1	Q08334, Q8IU57	2	112
2	P23458, P35568	2	109
3	Q06124, O60674	2	10
4	O15169, P49841, Q8N752, P17612, Q99835, O95996, P48730	7	67
5	P46531, P84022, P16220, Q13485	4	26

Proteins given by their UniProt ID. The table displays the number of proteins that can be affected by each dynamically positive SCC through positive interactions, thus staying activated if the given SCC stays in an autoactivated state. These are not independent: all SCCs can be reached from the first, all except the first can be reached from the second, and the fifth can be reached from the fourth (see also Fig. 2).

## Methods

### Metzler Matrices

Let 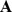, a square matrix, with elements 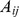, be a Metzler matrix, meaning that 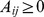 for all 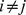, while the diagonal elements can be negative [16]. Let us consider the case when at least one element of the diagonal is negative. Let 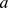 be the minimum of the diagonal: 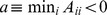. Then the matrix 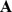 can be written in the form 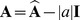, where 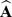 is nonnegative and 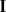 is the identity matrix. The matrices 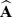 and 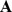 have the same eigenvectors with the corresponding eigenvalues only being shifted by 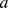. For the matrix 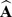 we can apply the Perron-Frobenius theorem [17] getting that 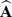 has a real largest eigenvalue (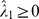 such that 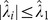 for all other eigenvalues) with the corresponding eigenvector having only nonnegative elements. For the original matrix 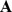, we have 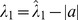, and for the real part of all other eigenvalues 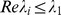. In the following sections we will refer to 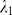 as the largest eigenvalue.

In the case of Eq. (2), the Jacobian matrix is a Metzler matrix: the diagonal elements are negative, while the off-diagonal elements 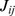 are positive when protein 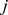 affects protein 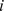, and zero otherwise. While in a general setting 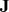 will not be symmetric, the Perron-Frobenius theorem guarantees that the eigenvalue with the largest real part will be real, and the corresponding 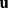 eigenvector can be chosen to have (real) nonnegative elements. In the case, when the signaling network is strongly connected (i.e. every node can be reached via directed links from every other node) the corresponding 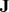 matrix will be irreducible. In this case the Perron-Frobenius theorem also states that 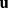 will have only positive elements [17].

### The Derivative of the Lyapunov Function

Let us consider Eq. (2) and presume that there exists a stationary solution 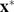 which is linearly stable. We will prove the existence of at least one such stationary state in the next section. Let 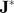 denote the Jacobian matrix computed at the stationary solution:
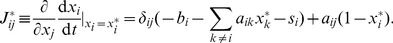
(5)


Linear stability means that all eigenvalues of 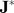 have negative real parts. A Lyapunov function can be constructed showing that for all initial conditions 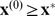 the system relaxes to the stationary solution 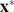. First let us rewrite Eq. (2) for new variables 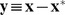:
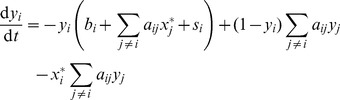
(6)


Notice that if 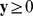 for some 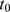 then 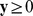 will hold for all 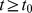. Since the solutions of Eq. (6) are continuous functions of 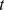, the system can only leave the 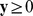 region if at some time 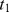, at least one component is zero. Substituting 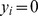 into Eq. (6) and assuming that 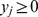 for all 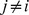, we will have a nonnegative derivate (recall that all 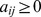 and 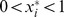):
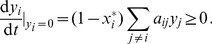
(7)


This allows us to define a Lyapunov function which is valid in the 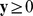 region (note that this will imply that our Lyapunov function is valid for all 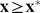, not just some neighborhood of it). The form of this Lyapunov function is:
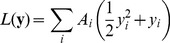
(8)where the 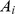 positive constants need yet to be chosen in an appropriate way. We have 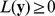 and 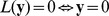. We need to choose the 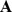 coefficients in a way which gives 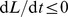 and 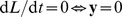. Computing the time derivative of 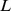 we get:
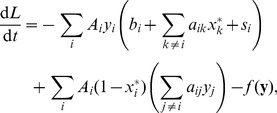
(9)where



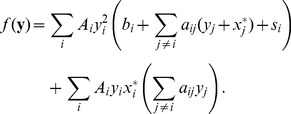
(10)For all 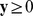, 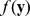 is nonnegative. This allows us to disregard 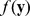 and only focus on the first part of the derivative, which can be written in a simpler way using the Jacobian matrix in the stationary solution defined in Eq. (5):
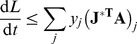
(11)where in parentheses the vector of coefficients 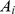 is multiplied by the transpose of the Jacobian matrix 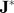. We get a negative result for all possible 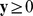 if all components are negative.

In the case of a strongly connected network, using that 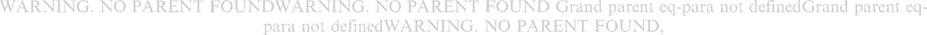 is a Metzler matrix, we get that the eigenvector corresponding to its largest eigenvalue can be chosen to have only positive elements (see the previous section). If the stationary solution 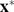 is linearly stable, the largest eigenvalue of 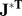 is negative. In this case, choosing the 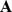 vector of coefficients to be the corresponding eigenvector, we have 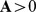 and 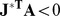. This choice gives a Lyapunov function which is valid for all 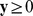.

In the case of a network with multiple SCCs, we can choose the 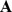 vector to be a linear combination of the eigenvectors of the submatrices corresponding to each separate strongly connected component. For this, let us reorder the 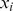 independent variables in such way, that the SCCs form blocks in 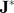. In a network with 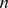 SCCs, we get a matrix with the following structure:
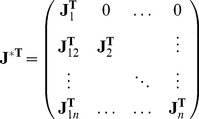
(12)


Here 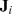 is the Jacobian matrix of the 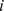-th SCC at the stationary solution 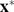 and 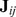 represents interaction between the 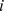-th and 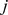-th SCC (e.g. the second SCC affects the first if 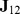 has nonzero elements). All elements above the diagonal blocks are zero (links between two distinct SCCs can be only in one direction). The stationary solution 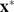 requires that all 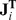 have only eigenvalues with negative real parts. Combining this with the fact that all 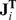 are now irreducible Metzler-matrices, we get that each submatrix will have a real largest eigenvalue 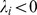 and corresponding real eigenvector 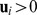. Now we can choose the 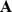 vector in Eq. (8) to be a “linear combination” of these eigenvectors: 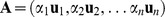. Of course we need to have 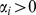 for all 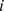. With this, we have:
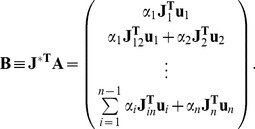
(13)


The first block in the resulting 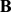 vector will be negative for any 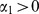. All other blocks can be separated into two terms with opposite sign:
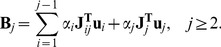
(14)


The summation will yield a nonnegative result, and the last term will be negative. Starting from 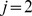 and heading consecutively to 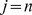, we can always choose 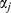 constants that are big enough for 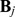 to be negative. In this way, we have constructed a suitable 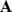 vector of coefficients for the Lyapunov function in Eq. (8).

Using the properties of the Lyapunov function, we get that for any initial condition 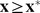 the system will converge to its stationary solution 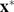. This also means that there can be at most one stable stationary solution; if there were more, their basins of attraction would overlap.

Note that our arguments presented for the networks with multiple SCCs do not actually require that we know the eigenvalues of 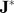; knowing that each submatrix 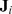 has only negative eigenvalues is sufficient. Of course, in a general setting the submatrices are not independent: 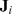 will be affected by 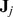 possibly for any 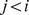. A rather special case is that when we have subsystems that all have stable off-states: any arrangement of these subsystems which does not include feedback loops will also have a stable off-state.

### The Lyapunov-function for the Off-state in the Presence of Inhibitory Interactions

Here we consider the special case of Eq. (3) when all 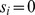 and the off-state is linearly stable (i.e. all eigenvalues of the Jacobian matrix are negative). We can define the Lyapunov function for this system in a way similar to the previous section:
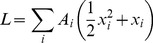
(15)where we require all 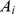 to be positive. Computing the time derivative we get a form similar to Eq. (9):
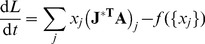
(16)where 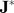 is the Jacobian matrix in the off-state, and 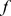 is nonnegative, and the inhibitory interactions only appear in 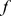. Using the same argument as in the previous section, we can choose an appropriate 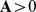 vector which will give a positive Lyapunov function and a negative time derivative. This means that the system will reach its stationary solution 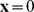.

### The Existence of a Stable Stationary Solution and Global Convergence

#### One SCC

Let us first consider a network that is strongly connected. In this case, the corresponding Jacobian matrix is irreducible. As all the off-diagonal elements are nonnegative, this implies that the dynamics of Eq. (2) is strongly monotone [18], [19]. This allows us to apply the Hirsch convergence theorem, which gives that the system converges to a stable stationary solution if started from any initial condition (except for a set with zero measure) [18]. Using the Lyapunov function defined in the previous sections, we can further state that there is exactly one stable stationary solution: each such solution 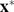 is attractive at least for initial conditions 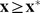, which means that multiple stable stationary solutions would have overlapping basins of attraction.

The Hirsch theorem is valid only for strongly connected systems; now we present an alternative proof of the existence and attractive properties of the stable stationary solution of the system in Eq. (2) which can be readily generalized for systems which consist of multiple SCCs.

For simplicity we will refer to the right-hand-side of Eq. (2) as 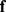 (with components 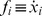). Notice that in the absence of an external signal (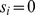), the origin is a stationary solution. If it is linearly stable, then the Lyapunov function (8) proves that it is globally attractive. Thus in the further analysis we will consider either the case when the origin is unstable (which we will refer to as case (i)) or that when there is an external signal (case (ii), note that now 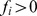 for at least one 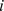 component). In these cases, we will first show that there is a nonzero stable stationary solution 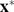 which can be connected to the origin with a path where 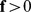. Next we can prove that for each 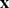 initial condition which is not itself an unstable stationary solution, the system will reach the 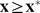 region where the Lyapunov function presented earlier is valid.

#### The existence of a stable stationary solution

We denote the Jacobian matrix at the origin by 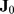. Multiplying the Jacobian matrix with a unit vector 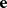 gives that how the components of 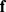 change, if we move in the direction pointed by 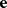 (i.e. 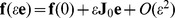).

In case (i), all derivatives in the origin are zero and the Jacobi matrix there has a real positive eigenvalue 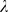 with a corresponding positive eigenvector 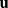. This implies that in the direction of 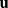, all components of 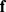 are increasing; as all 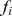 are continuous and infinitely differentiable, it follows that there is a set in the neighborhood of the origin, where 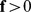.

In case (ii), at least one derivative 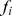 is positive at the origin (the others may be either positive or zero). In this case, we only need to find a direction where the zero components are increasing. This means that we need to find a positive vector 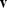 for which the vector 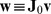 has 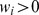 for values of 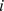, where 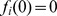 (the other components can be negative or zero in this case). Considering that 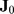 is a Metzler matrix, this is always possible.

Thus in both cases, we get that there is a set 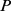 in the neighborhood of the origin, where all derivatives are positive:
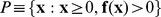
(17)


We further require that 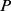 is connected and that the origin is an accumulation point of 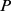 (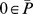). Now let us consider the closure of 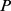, 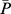, which contains points, where 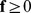. Note that both 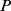 and 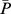 are bounded: 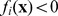 if 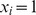. Let 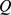 be the set of points in 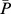 which are “farthest away” from the origin:
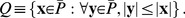
(18)


Now we can easily prove that every point in 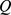 is a stable stationary solution. For this, let us consider a point 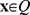. From the definition of 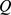, it follows, that there will be some points 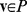 which are close to 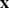. All these points must satisfy 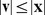. Now let us assume that 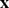 is not a stable stationary solution. This means that 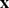 is either an unstable stationary solution or a point where some components of 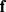 are positive, and some (or possibly none) are zero. In both cases, we will reach a contradiction by proving that there are some points 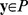 for which 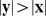. Using that the Jacobi matrix in 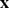 is a Metzler matrix and employing similar arguments as for 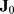, we can find a positive vector 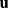 which points in a direction where again all components of 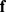 become positive. A more precise formulation of this is the following. Without the loss of generality, we can assume that the first 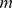 components of 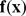 are zero, and the rest are positive (the order of the components is arbitrary). Of course, 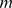 can be the total number of components in the case when 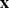 is assumed to be an unstable stationary solution. Using the very same arguments which we employed for the construction of the original 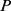 set, we have that 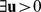, such that 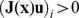 for all 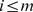. Using that 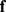 is a continuous and differentiable function of its variables, we have that 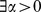 such that 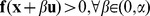. Now we have to employ the fact that 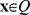. This means that there are points in 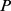 which are close to 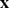: 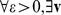 such that 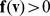, 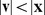 and 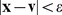 (of course, 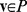). Furthermore, again using that 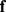 is continuous and differentiable, we have, that for small values of 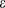, 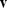 can arise as a linear approximation: 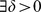 and 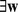 such that 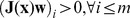, and also, 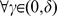 we have 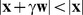 and 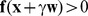 (i.e. we consider a line pointing from 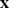 into 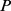).

Using these 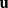 and 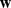 vectors, we can now prove that 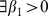 such that 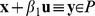. As 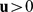, we will have 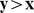, resulting in a contradiction. We have already seen that 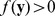 for the appropriate choice of 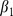; as we require 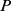 to be connected, we need to show that 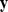 can be connected by a continuous curve to some other points known to be in 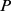. A such curve can be defined as 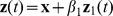, where.
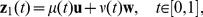
(19)and we require 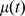 and 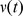 to be continuous functions with 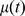 monotonically increasing and 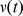 monotonically decreasing with boundary conditions 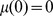, 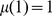, 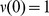 and 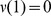. Notice that 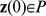, for small enough 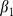. The derivatives along this curve will be:



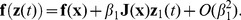
(20)Considering the second term, we have that 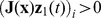, 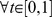, and 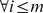 (these components are just the sum of two positive numbers). This implies that 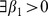 such that 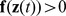, 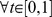. As 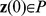, we have that 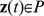, 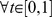 also. Applying this to 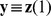 and using that 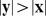, we have reached a contradiction: if 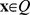, no such 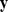 could exist (this was the definition of 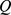). As our basic assumption was that 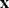 is not a stable stationary solution, it follows that 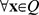 must be a stable stationary solution. Since 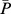 is bounded and nonempty, also 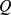 is nonempty, which means that there is at least one stable stationary solution. Applying the Lyapunov function presented previously, we get that there is exactly one stable stationary solution.

#### Convergence

Considering the 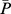 set defined above, we have seen that it contains two special points of interest: the origin and the stable stationary solution 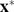 of the system. (both of these are accumulation points of the set 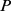). Using that the derivatives in Eq. (2) are all continuous and infinitely differentiable functions, it follows that we can construct a curve 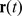, connecting the origin and 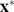 which is contained in the set 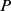:
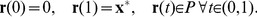
(21)


There are of course infinitely many such curves, but any one of them is suitable. We will refer to an arbitrary such curve as 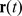. For every point on that curve, we will consider the following 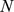-dimensional rectangle (if 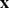 has 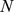 components):
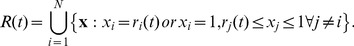
(22)


For any of these 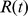 rectangles in the 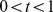 range, we have that the 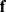 derivative vectors point toward the “inside”, in which the stable stationary solution resides. If any coordinate is 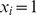, its derivative will be negative. On the other hand, if some coordinate is 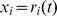, the corresponding derivative will be positive, since 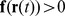 and the Jacobi matrix at any point has nonnegative off-diagonal elements. This implies that the solution of Eq. (2) is a flow, which heads into the 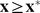 region, meaning that the system started from any initial condition will eventually reach this region, where the Lyapunov function defined earlier guarantees its convergence to 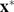.

#### Further remarks

The above proof about convergence holds for initial conditions where 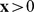. It is easy to see that our arguments are also valid for initial conditions where 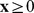 given that either there is an external signal or 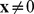 (we exclude the origin which is an unstable stationary solution if there is no external signal). In this case we have that at least one derivative 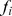 is positive. This means that there is a time 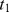 such that the corresponding coordinate 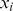 will be positive. That implies that for all 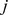, where 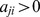, the 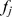 components will also be positive, meaning that at some time 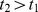 the 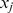 coordinates affected by 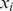 will be positive. Using that the network is strongly connected, and the derivatives are continuous and infinitely differentiable, we get that after some time 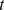, all components will be positive. Since we are in the 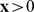 region now, the previous proof can be applied in this case too.

We also note that we have excluded the possibility when the largest eigenvalue of the Jacobian matrix at the origin is exactly zero. In this case our previous arguments do not hold. We think that in a real biological system this is not a concern; Eqs. (2) or (3) are valid only if the time scale of their solutions matches the time scale arising in other biological processes in the cell (i.e. a model based on ordinary differential equations is clearly unable to model the system if 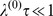, where 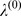 is the eigenvalue of the Jacobian matrix at a stationary solution and 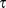 is the relevant timescale in the system).

#### More SCCs

We can inductively extend the proof presented in the previous section to a network which is not strongly connected, but is made up of an arbitrary combination of SCCs. Let us consider a network which is made up of two subnetworks, 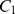 and 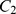, with links only in the 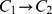 direction between them. We need to prove that if our results hold for both 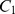 and 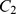 then they will also hold for the whole network. We can order the variables in a way that 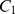 and 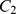 form blocks in 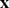. We will denote the variables belonging to each component with subscripts (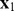 and 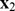 for the independent variables and 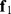 and 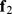 for the derivatives). Let us assume that our previous results hold for both components: for 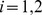, the system 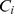 has a stable stationary solution in the presence of any stationary inputs. Also if this stationary solution is not the origin, then it can be connected to the origin with a path, where all derivatives are positive.

Let 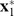 be the stable stationary solution of system 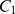. Using 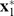 as constant inputs on system 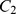, we can determine the 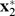 stable stationary solution of 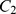. Now, the vector 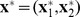 will be the stable stationary solution for the whole system.

Let us consider the Jacobian matrix in the origin, 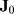, and also the submatrices corresponding to the two subsystem, 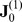 and 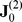. Let 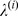 denote the largest eigenvalue of 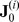. We have three separate cases:

- Case (1): 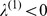 and 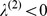. In this case 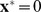, and the Lyapunov function proves its global attractive property.
- Case (2): 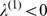 and 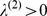. Now 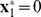 and 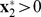.
- Case (3): 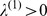. Here 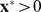.

Case (1) is covered by the Lyapunov function, so we have to give the proof only for cases (2)–(3).

**Case (2)** The component 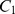 relaxes to its stationary solution. If we set 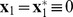, then the proof presented in the previous section is valid for component 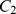. If we now set 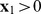, then all components of 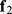 increase. Thus, the arguments presented for convergence in the previous section remain valid; the 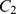 component will reach the 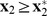 region. After that, for the whole system, we will have 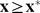, which again allows us to employ our Lyapunov-function, getting that the whole system converges to 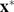.

**Case (3)** The proof of the global attractive properties is similar to the case of a strongly connected network, we only need to change some properties of the 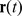 curve connecting the origin and the stable stationary solution. First we define the set 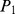 for the system 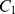 in a similar way as the set 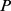 in the previous section (see Eq. (17)). We also define the set 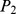 for system 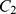 in this way with the restriction that we consider the system 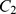 with constant inputs 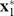. We consider the curve 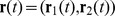, and require that:
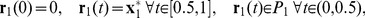
(23)

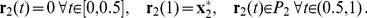
(24)


Again, we can use the continuously shrinking rectangles 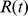 to prove that the system will reach the 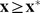 region and then converge to 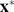.

**Figure 3:**
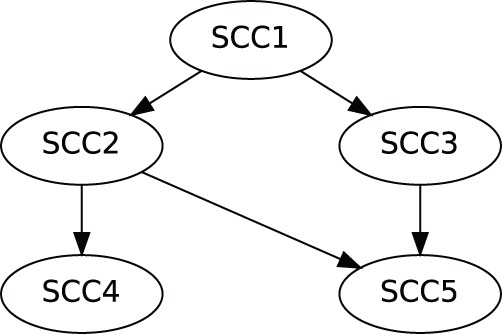
An example network. Here the bubbles represent SCCs, and the arrows represent links (and possibly cascade of nodes) between them. All nodes can have additional stationary inputs.

This way we get that if our previous results hold for systems 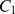 and 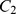, then they also hold for the combined system 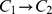. A network with more than two SCCs can always be built with the addition of one SCC at a time. Thus, for any network, we can start from an individual SCCs, and iteratively add each SCC, applying the arguments presented in this section at each step, getting that our results apply for the whole network. Considering the simple example network presented in Fig. 3., this means that we can prove our results for the whole network starting with 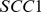 and consecutively adding 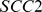, 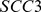, 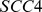 and 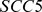.

## Discussion

We have shown, that the model suggested for the general treatment of signaling networks in [10] (Eq. (2)) always has one stationary solution, which is globally attractive (any initial condition relaxes to it), which means that the only relevant qualitative property of that model is that whether the off-state or an autoactivated state is the stable stationary solution in the absence of an external signal. This gives an example for a class of nonlinear systems which show only very simple behavior, and also an example for nonlinear systems where a Lyapunov function can be employed proving the attractive properties of a fixed point. This behavior is a consequence of the parameter space being bounded and the monotonic nature of Eq. (2), which gives a very simple flow as a solution. We have also shown that the straightforward generalization of the system to include inhibitory interactions (Eq. (3)) does not change the behavior in the case when the off-state is stable, thus the stability of the off-state can be determined by analyzing the network with only the positive interactions present.

While a more general model can have complex solutions, the observation that the stability of the off-state is determined by the behavior of the dynamically positive cycles in the network applies to a wider range of possible models. Counting such cycles in the Signalink database [4] of the human signaling network and also in randomly generated networks, we found that the real signaling network has significantly few dynamically positive cycles, which agrees with our expectations. This result supplements the findings of Kartal and Ebenhöh [12], who gained similar results for network data including only positive interactions.

Based on our findings, we expect a system transmitting an external signal to have a stable off-state which it returns to if the external system has been shut off. This should be the correct behavior in many cases. If, for some reason, this behavior changes the cell will “think” that there is an external signal when only the signal transmitting network is stuck in a faulty autoactivated state [12]. This could have drastic effects on the cell and cause it to cease carrying out its original purposes. The stability properties of the off-state could change following a change in the parameters of the system, caused by either the mutation of the proteins involved or the change in the protein concentrations. In the simple model considered here (Eq. (2) and (3)), this means a change in the matrix elements 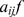 or 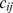. Computing the Jacobian matrix at the off-state and using the Perron-Frobenius theorem and the Collatz-Wielandt formula [17] we can see that the increase in the activation rate constants or the total protein concentrations causes an increase in the largest eigenvalue, and an increase in the deactivation rate constants causes the largest eigenvalue to decrease, while the inhibitory interactions 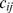 do not affect the linear stability. Mutations in certain proteins can thus lead to either the off-state of the network losing its stability giving a cell a constant stimulation as if it was constantly under an external signal, or to the dampening of the signal, lessening its effects on the cell (or even to the silencing of a pathway if a link is completely taken out). In our simple model a change in one of the parameter values can be compensated by changing some of the other parameters (e.g. the concentration of the phosphatases present). In a real network, more sophisticated methods will be needed, but we believe that our results concerning the importance of dynamically positive cycles can lead to a better understanding of intracellular signaling networks.

## References

[1] Barabási AL, Oltvai ZN (2004) Network biology: understanding the cell’s functional organization. Nat Rev Genet 5: 101–113. 14735121 10.1038/nrg1272

[2] Choi C, Krull M, Kel A, Kel-Margoulis O, Pistor S, et al (2004) Transpath – a high quality database focused on signal transduction. Comparative and Functional Genomics 5: 163–168. 18629064 10.1002/cfg.386 PMC2447348

[3] Gough NR (2002) Science’s signal transduction knowledge environment. Annals of the New York Academy of Sciences 971: 585–587. 12438188 10.1111/j.1749-6632.2002.tb04532.x

[4] Korcsmáros T, Farkas IJ, Szalay MS, Rovó P, Fazekas D, et al (2010) Uniformly curated signaling pathways reveal tissue-specific cross-talks and support drug target discovery. Bioinformatics 26: 2042–2050. 20542890 10.1093/bioinformatics/btq310

[5] Fazekas D, Koltai M, Türei D, Módos D, Pálfay M, et al (2013) SignaLink 2– a signaling pathway resource with multi-layered regulatory networks BMC Systems Biology. 7(1): 7. 10.1186/1752-0509-7-7 PMC3599410 23331499

[6] Tyson JJ, Chen KC, Novák B (2003) Sniffers, buzzers, toggles and blinkers: dynamics of regulatory and signaling pathways in the cell. Current opinion in cell biology 15: 221–231. 12648679 10.1016/s0955-0674(03)00017-6

[7] Levitzki A (1995) Signal transduction interception as a novel approach to disease management. Annals of the New York Academy of Sciences 766: 363–368. 7486682 10.1111/j.1749-6632.1995.tb26686.x

[8] Cui Q, Ma Y, Jaramillo M, Bari H, Awan A, et al (2007) A map of human cancer signaling. Molecular Systems Biology 3: 152. 18091723 10.1038/msb4100200 PMC2174632

[9] Copeland NG, Jenkins NA (2009) Deciphering the genetic landscape of cancer–from genes to pathways. Trends in genetics 25: 455–462. 19818523 10.1016/j.tig.2009.08.004

[10] Heinrich R, Neel BG, Rapoport TA (2002) Mathematical models of protein kinase signal transduction. Molecular Cell 9: 957–970. 12049733 10.1016/s1097-2765(02)00528-2

[11] Binder B, Heinrich R (2004) Interrelations between dynamical properties and structural characteristics of signal transduction networks. Genome Informatics 15: 13–23. 15712106

[12] Kartal O, Ebenhöh O (2009) Ground state robustness as an evolutionary design principle in signaling networks. PLoS ONE 4: e8001. 19956601 10.1371/journal.pone.0008001 PMC2779451

[13] Yi TM, Huang Y, Simon MI, Doyle J (2000) Robust perfect adaptation in bacterial chemotaxis through integral feedback control. Proceedings of the National Academy of Sciences 97: 4649–4653. 10.1073/pnas.97.9.4649 PMC18287 10781070

[14] Szabó A, Vattay G, Kondor D (2012) A cell signaling model as a trainable neural nanonetwork. Nano Communication Networks 3: 57–64.

[15] Sprott JC (2008) Simple models of complex chaotic systems. American Journal of Physics 76: 474–480.

[16] The Signalink Database. Available: http://www.signalink.org. Accessed April 15 2011.

[17] The Universal Protein Resource. Available: http://www.uniprot.org. Accessed February 14 2013.

[18] Mitkowski W (2008) Dynamical properties of metzler systems. Bulletin of the Polish Academy of Sciences, Technical Sciences 56: 309–312.

[19] Meyer CD, editor (2000) Matrix analysis and applied linear algebra, Chapter 8. Philadelphia, PA, USA: Society for Industrial and Applied Mathematics, 661–674 pp.

[20] Hirsch M, Smith H (2006) Chapter 4. In: A Canada PD, Fonda A, editors, Monotone Dynamical Systems, North-Holland, volume 2 of Handbook of Differential Equations: Ordinary Differential Equations. 239–357.

[21] Sontag E (2007) Monotone and near-monotone biochemical networks. Systems and Synthetic Biology 1: 59–87. 19003437 10.1007/s11693-007-9005-9 PMC2533521

